# Genomic Heterogeneity of *Cryptosporidium parvum* Isolates From Children in Bangladesh: Implications for Parasite Biology and Human Infection

**DOI:** 10.1093/infdis/jiad257

**Published:** 2023-10-13

**Authors:** Maureen Carey, Tuhinur Arju, James A Cotton, Masud Alam, Mamun Kabir, Abu S G Faruque, Rashidul Haque, William A Petri, Carol A Gilchrist

**Affiliations:** Departments of Medicine, University of Virginia, Charlottesville, Virginia, USA; International Centre for Diarrhoeal Diseases Research, Dhaka, Bangladesh; Wellcome Sanger Institute, Cambridge, United Kingdom; International Centre for Diarrhoeal Diseases Research, Dhaka, Bangladesh; International Centre for Diarrhoeal Diseases Research, Dhaka, Bangladesh; International Centre for Diarrhoeal Diseases Research, Dhaka, Bangladesh; International Centre for Diarrhoeal Diseases Research, Dhaka, Bangladesh; Departments of Medicine, University of Virginia, Charlottesville, Virginia, USA; Departments of Medicine, University of Virginia, Charlottesville, Virginia, USA

**Keywords:** *Cryptosporidium*, SNPs, genome sequences, parasite

## Abstract

*Cryptosporidium* species are a major cause of diarrhea and associated with growth failure. There is currently only limited knowledge of the parasite's genomic variability. We report a genomic analysis of *Cryptosporidium parvum* isolated from Bangladeshi infants and reanalysis of sequences from the United Kingdom. Human isolates from both locations shared 154 variants not present in the cattle-derived reference genome, suggesting host-specific adaptation of the parasite. Remarkably 34.6% of single-nucleotide polymorphisms unique to human isolates were nonsynonymous and 8.2% of these were in secreted proteins. Linkage disequilibrium decay indicated frequent recombination. The genetic diversity of *C. parvum* has potential implications for vaccine and therapeutic design.

**Clinical Trials Registration**. NCT02764918.

## Teaser

A genomic analysis of *C. parvum* isolated from Bangladeshi infants and reanalysis of sequences from the United Kingdom were performed. Full genome sequences clustered by *gp60* group and isolate source (human or animal) more than by geographic region.

*Cryptosporidium* parasites are a leading cause of death and disability due to diarrheal disease in infants in low- and middle-income countries [1–3]. The parasitic members of the eukaryotic single-celled *Cryptosporidium* genus can infect a broad range of hosts. While human cryptosporidiosis can be caused by at least 15 different species of *Cryptosporidium,* just 3 species cause the bulk of human disease: *Cryptosporidium hominis*, *Cryptosporidium meleagridis,* and *Cryptosporidium parvum* [4]. The aim of this work was to characterize the genetic diversity in the *Cryptosporidium* parasites infecting humans. No vaccine exists to prevent cryptosporidiosis and information on the population genetics of the parasite has important implications for the design and development of vaccines and therapeutics [5].

Fewer than 10 reference (formally assembled, annotated, and publicly available on the parasite database CryptoDB) genome sequences exist for these species [6]. Because only *C. parvum* can be cultured in vitro, *C. parvum* is the focus of many experimental models for vaccine and therapeutic development.

In previous work on cryptosporidiosis in Bangladesh and Africa, we and others discovered that extensive genomic diversity exists within *C. hominis,* including a high rate of sexual recombination and single nucleotide polymorphisms (SNPs) [7, 8]. Here, we extended this analysis by sequencing *C. parvum* isolates from children in Bangladesh and comparing these genome sequences to both the reference genome sequence, which was from cattle, and isolates collected from humans and cattle in the United Kingdom [9, 10]. We highlight the genomic variability among parasites collected in distinct geographic locations and between parasites isolated from humans versus cattle, as well as a high rate of recombination. These findings emphasize the need for further delineation of the variability in clinically relevant reference genome sequences to prioritize antigen selection and identify drug targets.

## METHODS

### Ethical Considerations

The study was approved by the Ethical and Research Review Committees of the International Centre for Diarrhoeal Disease Research, Bangladesh (icddr, b) and the Institutional Review Board of the University of Virginia. Informed written consent was obtained from the parents or guardians for the participation of their child in the study.

### Infant Cohort

Starting in June 2014, 250 children born into an urban slum of Dhaka (Section 11 of Mirpur Thana) and 258 children from rural Mirzapur were enrolled in the first week after birth into a community-based prospective cohort study of enteric infections (“Cryptosporidiosis and Enteropathogens in Bangladesh”; ClinicalTrials.gov identifier NCT02764918). At the urban location, an additional 250 children from the same population were enrolled in a second companion community-based prospective cohort study focused on infant cryptosporidiosis (“Field Studies of Cryptosporidiosis and Enteropathogens in Bangladesh”). *C. parvum* was identified in 2% of all cryptosporidia infections (n = 3, 2 monoinfections) at the urban site and in 4% (total n = 4; 3 monoinfections) at the rural location [4]. Six of the oocyst isolates collected from both study sites had sufficient DNA for sequencing and were included in the analysis reported here, including 1 coinfected with *C. hominis*.

### Sampling and Specimen Testing

Diarrheal and monthly surveillance stools were evaluated for the presence of the major enteric parasitic pathogens, including *Cryptosporidium* spp. by use of a multiplexed quantitative polymerase chain reaction (qPCR) assay, which includes a broad-range assay that recognizes the common *Cryptosporidium* species that routinely infect humans (*C. hominis*, *C. parvum,* and *C. meleagridis*) as well as 2 other clinically relevant protozoan parasites (*Giardia duodenalis* and *Entamoeba histolytica*) [7, 11].

### Genotyping Assay

The polymorphic region within the *gp60* gene (cgd6_1080) was used to genotype *Cryptosporidium*-positive samples [12]. If the short-read genomic sequences were insufficient to type the region with confidence, a nested PCR reaction was performed as previously described and Sanger sequenced [7] (Supplementary Table 1**)**. Mixed infections were identified by the presence of multiple *gp60* genotypes.

### Whole-Genome Resequencing and Analysis

The sequences of the Bangladesh isolates were obtained as previously described for *C. hominis* [7] and are deposited in the NCBI's Sequence Read Archive BioProject PRJEB14327 (SRA; Supplementary Table 1). Additional genome sequences were obtained from the SRA with the following selection criteria: availability of fastq files and sample collection methods presented in peer-reviewed publications. Furthermore, only samples that were collected from humans and purified to obtain oocysts were used, excluding mouse-derived parasites and metagenomic sequences. With these selection criteria, whole-genome nucleotide sequencing data from 12 isolates collected in the United Kingdom were acquired through their accession numbers (Supplementary Table 1) [9, 13]. Of note, 1 additional genome sequence (from a child in Uganda) passed these selection criteria; it was excluded because, with only 1 isolate, we could not compare multiple Ugandan isolates as we did with isolates from the United Kingdom and Bangladesh [7, 9].

For consistency in analysis, all genome sequences were analyzed in parallel and aligned to the *Cryptosporidium parvum* Iowa II reference genome sequence (CryptoDB version 53; [6]); the analytic code and additional detail are provided at https://github.com/maureencarey/cparvum_genomes_manuscript. First, sequences were downloaded from NCBI's SRA and trimmed to remove adaptors and restrict sequence length to 150 base pairs, and quality filtered with BBTools version 38.57 [14]. Read quality was evaluated with FastQC version 0.11.5 and MultiQC version 1.8 [15, 16]. Next, unmerged forward and reverse reads were aligned with BWA-mem version 0.7.17 [17] to the *C. parvum* Iowa II reference genome (accession number GCA_000165345.1), SAM files were converted and sorted to generate BAM files with SAMTools version 1.9 [18]. The mean depth of genome cover in each isolate was determined using (SAMTools; version 1.12) and multiallelic SNPs were identified using Freebayes (version 0.9.9) and tabulated using bcftools (version 1.9). Duplicate reads were marked with Picard version 2.20.6 (https://broadinstitute.github.io/picard). GATK's HaplotypeCaller (version 4.0.0.0) was then used to call SNPs [19]. For the combined population-based analyses the individual BAM files were then merged (Supplementary Table 1**)**. For SNP analyses, oocyst genome sequences were treated as tetraploid; however, for linkage disequilibrium calculations, these genome sequences were treated as diploid for compatibility with available software [20]. Lastly, variants were filtered to remove low-quality SNPs using GATK's VariantFiltration (QUAL < 25.0, QD < 15.0, FS > 12.0, MQ < 58.0, MQRankSum < −3.0, ReadPosRankSum < −3.0 [19]); parameters were selected for consistency with our previous publication [7]. SnpEff version 4.3 was used to identify the variations that resulted in the substitution of a different amino acid in the encoded proteins [21].

Linkage disequilibrium and principal component analysis (PCA) were performed with PLINK version 1.90b6.16 [20, 22]. VCF files were analyzed and visualized in R 4.0.3 [23, 24]. The 32 Bangladesh *C. hominis* genome sequences [7] were rerun through an identical pipeline for comparison and randomly subset into groups of 16–18 to determine if the differences in the squared correlation coefficient between different alleles (*r*^2^) resulted from sample size bias [25].

## RESULTS

**Figure 1.**
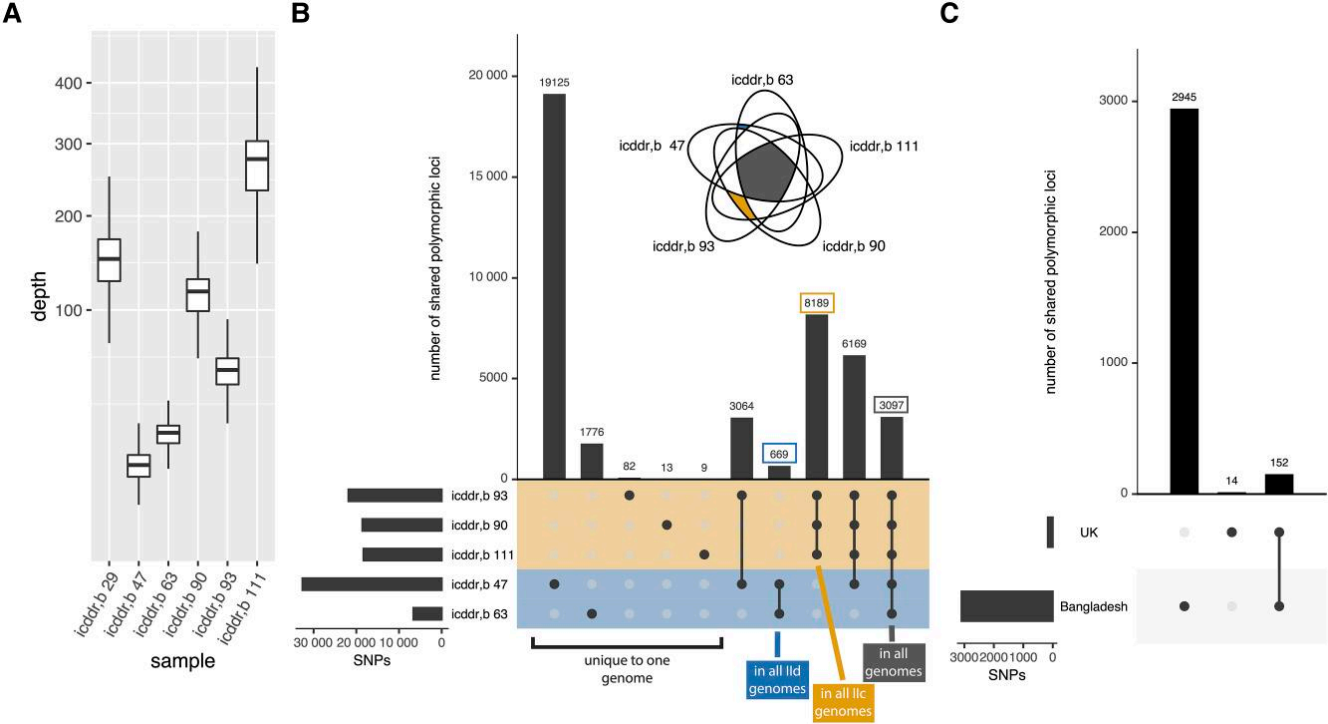
Novel genome sequencing reveals conserved variants and variants associated with geographic location. *A*, Sequencing depth. Genome-wide sequencing depth for each Bangladesh isolate (including the mixed infection); analogous data for UK isolates is available in Supplementary Figure 1. Each box represents the median (inner line), 25th percentile, and 75th percentile. Upper whiskers extend from the top of the box to the largest value within 1.5 times the interquartile range, and the lower whisker extends to the smallest value within 1.5 times the interquartile range. *B*, Core set of conserved single-nucleotide polymorphisms (SNPs) and SNPs unique to each genome. SNPs in each monoinfection isolate from Bangladesh were compared using the UpSet visualization approach. Left barchart represents the total number of SNPs in each genome. Top bar chart represents the number of SNPs shared between the different genome sequences (indicated by the filled circles below). The mixed infection is not shown. Genome sequences in yellow are IIc (*gp60* grouping), genome sequences in blue are IId. The inset Venn diagram is shown to highlight the SNPs shared between the genome sequences with the different *gp60* genotype. *C*, SNPs found in all isolates. SNPs found in all Bangladesh isolates or all UK isolates were compared.

Oocysts purified by immunomagnetic separation from stool were sequenced to obtain 5 *C. parvum* genome sequence monoinfections (as determined by unique polymorphic *gp60* region and only a small number of multiallelic SNPs [0.1% ± 0.08%]) and the mean depth of cover (DP) and the percentage of the reads that mapped to the *C. parvum* genome (mapped) for each isolate calculated (icddr, b 47, DP = 20.6, mapped 58.85%; icddr, b 63, DP = 32.25, mapped =97.97%; icddr, b 90, DP = 142.259, mapped = 88.74%, icddr, b 93, DP = 83.23, mapped = 88.32%; icddr, b 111, DP = 263.126, mapped = 99.40%). One additional isolate was also obtained from a child with a mixed *C. parvum* and *C. hominis* infection (icddr, b 29: DP = 194, mapped = 98.35%) (Figure 1A, Supplementalry Table 1). Parasite genome sequences contained between 6636 and 32 616 SNPs (total, 43 882) when compared to the reference genome, *C. parvum* Iowa II obtained from cattle (CryptoDB version 53) (Figure 1). No major structural variations (defined as insertions or deletions greater than 200 base pairs) were observed (see code, Supplementary Material). Only 3097 SNPs were common to all 5 monoinfection isolates (Figure 1*B*). For reference, 12 recently published genome sequences isolated in the United Kingdom sequenced using similar technology to a mean coverage of 101.5 reads per locus, were analyzed in parallel (Supplementary Figure 1*A*). In the UK isolates, 26 992 SNPs were detected, but only 166 of these SNPs were present in all of the UK isolates (Supplementary Figure 1*B*). There were 2945 SNPs found exclusively in the Bangladesh isolates (ie, in all Bangladesh isolates and in no UK isolates) whereas only 14 SNPs were found exclusively in the UK isolates (Figure 1*C*). There were 152 SNPs found to be shared with all of the human isolates, including 60 variants that resulted in a change in the amino acid sequences of the encoded proteins. Despite variability in the total number of SNPs and genes affected by SNPs across genome sequences (Supplementary Figure 2*A* and 2*B*), nonsynonymous variants were similarly represented in each genome (Supplementary Figure 2*C*); nonsynonymous SNPs yield functional differences in a protein by changing the amino acid sequence or adding or removing a stop codon. Such changes in the amino acid sequence in secreted proteins were of particular interest given the potential for interaction with the mammalian host and, therefore, these may be under selective pressure. Ninety-five genes in the *C. parvum* genome are predicted to encode secreted proteins (2.4% of all genes [6]). We found that the nonsynonymous SNPs occurred at a higher frequency in these genes (3% of the nonsynonymous SNPs were located in these genes) (Supplementary Figure 2*C*). Five genes encoding secreted proteins contained SNPs in all of these human isolates: cgd8_1740 (secreted GGC gene family protein), cgd3_10 (uncharacterized, SKSR gene family), cgd6_1180 (uncharacterized), cgd7_4340 (uncharacterized), cgd7_4500 (uncharacterized), and cgd8_3540 (uncharacterized, WYLE gene family). Three genes contained SNPs in only the Bangladesh isolates: cgd1_1680 (insulinase-like protease), cgd7_3390 (patatin-like phospholipase), and cgd8_3670 (uncharacterized); none of these proteins are known to trigger a host immune response.

**Figure 2.**
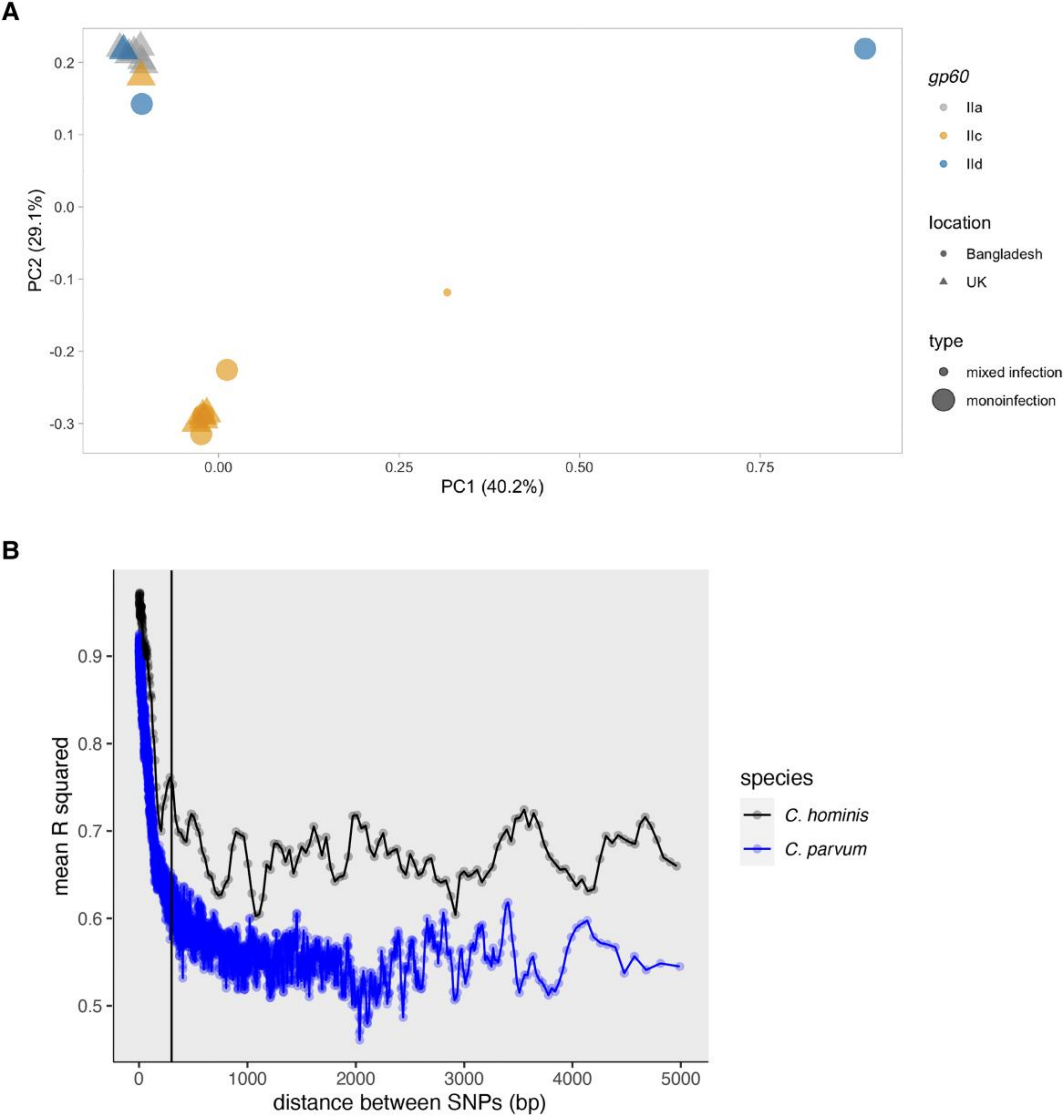
Despite the rapid linkage disequilibrium decay observed in the *Cryptosporidium parvum* genome sequences the whole-genome SNP profile continued to be concordant with the *gp60* genotype. *A*, *gp60* summarizes genome-wide variation. Principal component (PC) analysis of genome-wide variation (as implemented in Plink 2.0) is shown. All quality-filtered single-nucleotide polymorphisms (SNPs) were used. Points represent genome sequences and are color coded by *gp60* grouping. Shapes represent patient location. The small dot represents the mixed infection from Bangladesh. Significance as determined by a PERMANOVA: location, *P* > .5; *gp60*, *P* < .04; however, this statistical test is biased by outliers, and so please see Supplementary Figure 3. *B*, Similar rate of recombination in *C. parvum* when compared to *Cryptosporidium hominis.* Comparison between *C. parvum* (blue) and *C. hominis* (black) linkage disequilibrium decay, calculated with Plink 2.0. The region of linkage disequilibrium in *C. hominis* was previously calculated as being <300 and 300 bp is indicated by the vertical black line at 300 bp on the x-axis [7]. Although the threshold values were different in *C. hominis* and *C. parvum* the rate of linkage disequilibrium decay appeared similar. To confirm this observation the data were reanalyzed using comparable genome numbers (Supplementary Figure 4).

By comparing all SNP variations in each genome using a PCA, the full genome sequences clustered by *gp60* group more so than geographic region (PERMANOVA, location *P* > .5; *gp60 P* < .04; Figure 2*A*). *gp60* is the most frequently used *Cryptosporidium* genotyping system. The *gp60* locus is evaluated by identifying individual SNPs to distinguish parasite types and the number of repetitions in a highly variable microsatellite region to identify subtypes [26,27]; the *C. parvum gp60* group IIc is thought to have a strong preference for human hosts whereas *C. parvum* isolates with other *gp60* genotypes are thought to more commonly infect other mammals [9]. The reference genome from *C. parvum* Iowa II, used in this study, belongs to the IIa group and therefore would be expected to have a broad range of mammalian hosts (GCA_015245375.1 VEupath Release 53) [28]. This trend in *gp60* group clustering via PCA was conserved when focusing on only those nonsynonomous SNPs that resulted in changes in the amino acid sequences of the secreted proteins (Supplementary Figure 3*A* and 3*B*). Because PCA and associated statistical tests can be biased by outliers, we also confirmed that this trend was consistent when excluding 1 genome from Bangladesh that appeared to be an outlier (identifier icddr, b 47). Outlier status was based on SNP content after removing biases (base pairs were filtered for quality and normalized to read depth and root mean square mapping quality over all the reads at the site). The reanalysis via PCA of the data minus icddr, b 47 (Supplementary Figure 3*C*) did not alter our initial conclusion based on the number of shared SNPs (Figure 1*B*) or our conclusion after the performance of the first PCA that the genome sequences belonging to the *gp60* group IIc in the United Kingdom and Bangladesh were more similar to each other than to the group IId genome sequences (Figure 2*A*).

Previous work highlighted the unusually short regions of linkage disequilibrium in *C. hominis* (<300 bp) and, as a result, *C. hominis* genome sequences do not cluster by *gp60* genotype [7]. In *C. parvum* the *gp60* genotype is, however, routinely used to both track outbreaks and as a genetic marker to describe genomic lineages [9, 12]. Its use for this purpose has, however, been controversial [29–32]. Thus, we next asked if *gp60* was more representative of whole-genome *C. parvum* diversity in Bangladesh than in *C. hominis* because *C. parvum* has less recombination. Our results, however, indicated that this is not the case. The *C. parvum* parasites also had a high rate of recombination (Figure 2*B*) similar to that occurring in the Bangladesh *C. hominis* genome sequences [25]. Any differences in observed linkage disequilibrium curves are explained by the reduced number of genome sequences included in the study when compared to the *C. hominis* study (Supplementary Figure 4) [25].

## DISCUSSION

Here, we demonstrate that *C. parvum* isolates from human infections in geographically diverse locations have genetic differences from the reference genome line, isolated from cattle. We present high-quality sequence data from 5 *C. parvum* isolates collected from patients in Bangladesh (Figure 1*A* and 1*B*), compared them to both the animal-derived reference genome (*C. parvum* Iowa II) and 12 recently published human isolates from the United Kingdom, and identified a set of SNPs shared by all 17 human isolates (Figure 1*C*). Over 39% of these SNPs specific to *Cryptosporidium* genome sequences isolated from human hosts resulted in functional differences in the encoded proteins; these variants highlight key differences between human-derived isolates and the animal-derived parasite line that was used to generate a reference genome. Importantly, 3% of all SNPs in the Bangladesh and UK isolates were both nonsynonymous and located within the 2.4% of genes that encode for secreted proteins, which are potentially under selection pressure from the human host (Supplementary Figure 2).

With only 2 publicly available *C. parvum* reference genome sequences [6] and fewer than 10 published whole-genome sequencing studies, this study adds considerably to the field of parasite genomics by increasing the number and diversity of available genome sequences, specifically those derived from human infections. As such, 4 of these novel genome sequences obtained from monoinfections were *gp60* group IIc, the genotype group described as the human *C. parvum* (*C. parvum anthroponosum*; Figure 1*B*). Importantly, the Bangladesh isolates had more total SNPs and more shared SNPs than the UK isolates (Figure 1*B* and Supplementary Figures 1*B* and 2*A*), emphasizing the diversity of parasites in this region [7].

Furthermore, we show that *gp60* is somewhat representative of full genomic diversity in *C. parvum* in this set of genome sequences. Genome sequences with the same *gp60* genotype are more similar than genome sequences from a single geographic location (Figure 1*B* and Figure 2*A*). The (partial) utility of *gp60* genotyping in this species is contrary to what was observed in *C. hominis* [7]. Lastly, we show that this difference between species is not due to a significantly decreased rate of recombination in *C. parvum* (Figure 2*B*). The species difference may be the result of differential selective pressure on the gp60 protein in each species. As the function of gp60 is uncharacterized and the host range varies between the 2 parasite species, functional differences across species in the protein or in the host's response to the protein are plausible.

This study has several limitations, most notably the small number of *C. parvum* genome sequences obtained from human and cattle samples and the limited geographic range of infection locations sampled. However, the study also has notable strengths including the acquisition of the *C. parvum* genome sequences isolated from a Bangladesh population where this parasite is endemic and the identification of a core set of SNPs apparently unique to human isolates of *C. parvum*.

## CONCLUSIONS

Clinically derived isolates of *C. parvum* collected in Bangladesh were highly divergent from one another with over 30% of SNPs in these isolates resulting in changes in protein open reading frames. Genetic variability within this population is unexpected given the low prevalence of this *Cryptosporidium* species and the homogenous host population [4]. Furthermore, frequent recombination also occurred in *C. parvum*, consistent with a previous study on *C. hominis.* Unlike *C. hominis*, however, the *gp60* genotyping system remained representative of genomic variation in *C. parvum.* Bangladeshi human-derived isolates share many SNPs with clinically derived isolates from the United Kingdom, indicating divergence from the animal-derived reference genome and emphasizing the need for high-quality reference genome sequences from both human and animal infections. The functional significance of these genomic changes in the parasite remains to be discovered, but highlights that such genetic diversity may need to be accounted for in identifying targetable antigens for vaccine development.

## Supplementary Data

Supplementary materials are available at *The Journal of Infectious Diseases* online. Consisting of data provided by the authors to benefit the reader, the posted materials are not copyedited and are the sole responsibility of the authors, so questions or comments should be addressed to the corresponding author.

## Supplementary Material

jiad257_Supplementary_Data

## Notes

***Author contributions.*** T. A. processed samples and J. A. C. provided sequencing data. M. A. C., C. A. G., and W. A. P. conceived the analysis plan and M. A. C. performed bioinformatic analyses with J. A. C. providing consultation. W. A. P., R. H., and A. S. G. F. founded the birth cohort and directed the study. Field work and data collection at the International Centre for Diarrhoeal Disease Research, Bangladesh (icddr, b) were performed by M. A. and M. K., with supervision from A. S. G. F. and R. H. Drafting of the manuscript was performed by M. A. C. and C. A. G. All authors edited and approved the final manuscript.

***Acknowledgement.*** We are grateful to Dr Matthew Berriman for advice and assistance in the sequencing and for the willing participation of the parents and children at the icddr, b study sites. We also thank the field workers, nurses, laboratory staff of the Parasitology Laboratory of icddr, b who worked for this project, and Gladys Andino, at the University of Virginia Research Computing Core without whom we could not have completed this research. We also acknowledge the helpful feedback from members of the Petri laboratory, University of Virginia Trans-University Microbiome Initiative's Data Science team, and Pankaj Kumar, as well as Uma Nayak for her curation of the clinical database.

***Disclaimer.*** The funders had no role in study design, data collection and analysis, or decision to submit for publication.

***Financial support.*** This work was supported by the National Institute of Allergy and Infectious Diseases (grant numbers R21 AI-109118 to C. A. G., and R01 AI-043596 to C. A. G. and W. A. P.); the Bill and Melinda Gates Foundation (grant number OPP1100514 to A. S. G. F.); University of Virginia Engineering-in-Medicine seed grant to M. A. C. and W. A. P.; the Henske Family Foundation; and the Wellcome Trust (grant number 206194). M. A. C. was supported by the PhRMA Foundation Postdoctoral Fellowship in Translational Medicine and Therapeutics and the University of Virginia Trans-University Microbiome Initiative. TechLab, Inc donated the *Giardia*/*Cryptosporidium* QUIK CHEKS used in the parent study. Work at icddr, b was supported by the core donors (Government of the People’s Republic of Bangladesh, Global Affairs Canada, the Swedish International Development Cooperation Agency, and UKAid).

***Potential conflict of interests.*** W. A. P. is a consultant for TechLab, a company that makes diagnostic tests for cryptosporidiosis. All other authors report no potential conflicts.

## References

[1] Haque R , Mondal D, Karim A, et al Prospective case-control study of the association between common enteric protozoal parasites and diarrhea in Bangladesh. Clin Infect Dis 2009; 48:1191–7. 19323634 10.1086/597580 PMC2883291

[2] Liu J , Platts-Mills JA, Juma J, et al Use of quantitative molecular diagnostic methods to identify causes of diarrhoea in children: a reanalysis of the GEMS case-control study. Lancet 2016; 388:1291–301. 27673470 10.1016/S0140-6736(16)31529-X PMC5471845

[3] Platts-Mills JA , Babji S, Bodhidatta L, et al Pathogen-specific burdens of community diarrhoea in developing countries: a multisite birth cohort study (MAL-ED). Lancet Glob Health 2015; 3:e564–75. 26202075 10.1016/S2214-109X(15)00151-5 PMC7328884

[4] Steiner KL , Ahmed S, Gilchrist CA, et al Species of cryptosporidia causing subclinical infection associated with growth faltering in rural and urban Bangladesh: a birth cohort study. Clin Infect Dis 2018; 67:1347–55. 29897482 10.1093/cid/ciy310 PMC6186860

[5] Sparks H , Nair G, Castellanos-Gonzalez A, White AC. Treatment of *Cryptosporidium*: what we know, gaps, and the way forward. Curr Trop Med Rep 2015; 2:181–7. 26568906 10.1007/s40475-015-0056-9 PMC4640180

[6] Heiges M . CryptoDB: a *Cryptosporidium* bioinformatics resource update. Nucleic Acids Res 2006; 34:D419–22. 16381902 10.1093/nar/gkj078 PMC1347441

[7] Gilchrist CA , Cotton JA, Burkey C, et al Genetic diversity of *Cryptosporidium* hominis in a Bangladeshi community as revealed by whole-genome sequencing. J Infect Dis 2018; 218:259–64. 29514308 10.1093/infdis/jiy121 PMC6009673

[8] Tichkule S , Jex AR, van Oosterhout C, et al Comparative genomics revealed adaptive admixture in *Cryptosporidium hominis* in Africa. Microb Genom 2021; 7:mgen000493. 10.1099/mgen.0.000493 PMC8115899 33355530

[9] Nader JL , Mathers TC, Ward BJ, et al Evolutionary genomics of anthroponosis in *Cryptosporidium*. Nat Microbiol 2019; 4:826–36. 30833731 10.1038/s41564-019-0377-x

[10] Abrahamsen MS , Templeton TJ, Enomoto S, et al Complete genome sequence of the apicomplexan, *Cryptosporidium parvum*. Science 2004; 304:441–5. 15044751 10.1126/science.1094786

[11] Liu J , Kabir F, Manneh J, et al Development and assessment of molecular diagnostic tests for 15 enteropathogens causing childhood diarrhoea: a multicentre study. Lancet Infect Dis 2014; 14:716–24. 25022434 10.1016/S1473-3099(14)70808-4

[12] Chalmers RM , Robinson G, Elwin K, Elson R. Analysis of the *Cryptosporidium* spp. and *gp60* subtypes linked to human outbreaks of cryptosporidiosis in England and Wales, 2009 to 2017. Parasit Vectors 2019; 12:95. 30867023 10.1186/s13071-019-3354-6 PMC6417012

[13] Hadfield SJ , Pachebat JA, Swain MT, et al Generation of whole genome sequences of new *Cryptosporidium hominis* and *Cryptosporidium parvum* isolates directly from stool samples. BMC Genomics 2015; 16:650. 26318339 10.1186/s12864-015-1805-9 PMC4552982

[14] Bushnell B .BBMap: BBTools: a suite of fast, multithreaded bioinformatics tools designed for analysis of DNA and RNA sequence data. https://sourceforge.net/projects/bbmap/. Accessed 8 August 2022.

[15] Ewels P , Magnusson M, Lundin S, Käller M. MultiQC: summarize analysis results for multiple tools and samples in a single report. Bioinformatics 2016; 32:3047–8. 27312411 10.1093/bioinformatics/btw354 PMC5039924

[16] 08/2022Andrews S . FastQC: a quality control tool for high throughput sequence data. Babraham Bioinformatics, Babraham Institute, 2010. https://www.bioinformatics.babraham.ac.uk/projects/fastqc/. Accessed 8 August 2022.

[17] Li H , Durbin R. Fast and accurate short read alignment with Burrows-Wheeler transform. Bioinformatics 2009; 25:1754–60. 19451168 10.1093/bioinformatics/btp324 PMC2705234

[18] Li H , Handsaker B, Wysoker A, et al The sequence alignment/map format and SAMtools. Bioinformatics 2009; 25:2078–9. 19505943 10.1093/bioinformatics/btp352 PMC2723002

[19] Poplin R , Ruano-Rubio V, DePristo MA, et al Scaling accurate genetic variant discovery to tens of thousands of samples. bioRxiv, doi: 10.1101/201178, 24 July 2018, preprint: not peer reviewed.

[20] Chang CC , Chow CC, Tellier LC, Vattikuti S, Purcell SM, Lee JJ. Second-generation PLINK: rising to the challenge of larger and richer datasets. Gigascience 2015; 4:7. 25722852 10.1186/s13742-015-0047-8 PMC4342193

[21] Cingolani P , Platts A, Wang LL, et al A program for annotating and predicting the effects of single nucleotide polymorphisms, SnpEff. Fly (Austin) 2012; 6:80–92. 22728672 10.4161/fly.19695 PMC3679285

[22] Purcell S , Neale B, Todd-Brown K, et al PLINK: a tool set for whole-genome association and population-based linkage analyses. Am J Hum Genet 2007; 81:559–75. 17701901 10.1086/519795 PMC1950838

[23] RStudio Team . RStudio: integrated development for R. https://posit.co/download/rstudio-desktop/. Accessed 7 August 2022.

[24] Wickham H , Chang W, Henry L, et al ggplot2: Create Elegant Data Visualisations Using the Grammar of Graphics. CRAN R-Project. http://CRAN.R-Project.Org/package=ggplot2.

[25] Tenesa A , Navarro P, Hayes BJ, et al Recent human effective population size estimated from linkage disequilibrium. Genome Res 2007; 17:520–6. 17351134 10.1101/gr.6023607 PMC1832099

[26] Cama VA , Bern C, Roberts J, et al *Cryptosporidium* species and subtypes and clinical manifestations in children, Peru. Emerg Infect Dis 2008; 14:1567–74. 18826821 10.3201/eid1410.071273 PMC2609889

[27] Baptista RP , Li Y, Sateriale A, et al Long-read assembly and comparative evidence-based reanalysis of *Cryptosporidium* genome sequences reveal expanded transporter repertoire and duplication of entire chromosome ends including subtelomeric regions. Genome Res 2022; 32:203–13. 34764149 10.1101/gr.275325.121 PMC8744675

[28] Tanrıverdi S , Grinberg A, Chalmers RM, et al Inferences about the global population structures of *Cryptosporidium parvum* and *Cryptosporidium hominis*. Appl Environ Microbiol 2008; 74:7227–34. 18836013 10.1128/AEM.01576-08 PMC2592928

[29] Widmer G , Lee Y. Comparison of single- and multilocus genetic diversity in the protozoan parasites *Cryptosporidium parvum* and *C. hominis*. Appl Environ Microbiol 2010; 76:6639–44. 20709840 10.1128/AEM.01268-10 PMC2950454

[30] Robinson G , Chalmers RM. Assessment of polymorphic genetic markers for multi-locus typing of *Cryptosporidium parvum* and *Cryptosporidium hominis*. Exp Parasitol 2012; 132:200–15. 22781277 10.1016/j.exppara.2012.06.016

[31] Abal-Fabeiro JL , Maside X, Bello X, Llovo J, Bartolomé C. Multilocus patterns of genetic variation across *Cryptosporidium* species suggest balancing selection at the *gp60* locus. Mol Ecol 2013; 22:4723–32. 23915002 10.1111/mec.12425

[32] Afgan E , Nekrutenko A, Grüning BA, et al The galaxy platform for accessible, reproducible and collaborative biomedical analyses: 2022 update. Nucleic Acids Res 2022; 50:W345–51. 35446428 10.1093/nar/gkac247 PMC9252830

