# Supplementary material for: Genomic Heterogeneity of Cryptosporidium parvum Isolates From Children in Bangladesh: Implications for Parasite Biology and Human Infection
Source: J Infect Dis. 2023 Oct 13;228(9):1292–8. doi: 10.1093/infdis/jiad257 (PMC10629705; doi:10.1093/infdis/jiad257)
Supplement: jiad257_Supplementary_Data [file jiad257_supplementary_data.zip › Supplemental Figure Legends.docx]

**Supplemental Figures**

**Supplemental Figure 1: A: Sequencing depth of previously published genomes.** Genome-wide sequencing depth for all samples, including UK isolates. Each box represents the median (inner line), 25th percentile and 75th percentile. Upper whiskers extend from the top of the box to the largest value within 1.5 times the interquartile range (distance between 25th and 75th percentile), and the lower whisker extends to the smallest value within 1.5 times the interquartile range. The UK isolates with <5000 SNPs are designated as *C. parvum parvum* by Nader et al (5) **B: Core conserved SNPs in all isolates from the UK**. Comparison of the SNPs for each isolate from the UK. Top bar chart represents the number of SNPs shared by genomes (indicated below); this plot can be interpreted similar to a Venn diagram. SNPs associated with just one genome are shown in the first five bars with the largest intersections shown only in the remaining four bars. The mixed infection is not shown. Left bar chart represents the total number of SNPs in each genome. Genomes highlighted in yellow bar (bottom) are IIc (gp60 grouping), blue is IId, and grey are IIa.

**Supplemental Figure 2: SNPs with functional consequences.** Total number of SNPs (**A**) or genes with SNPs (**B**) per genome. Grey bar represents total number, and red represents the number of nonsynonymous SNPs. Dark red is used to highlight nonsynonymous SNPs in genes that encode secreted proteins. **C:** Violin plot showing the fraction of SNPs with functional consequences in each genome. X-axis genomic SNPs Y-axis indicates the % of SNPs with nonsynonymous or synonymous variants. Red dots the Bangladesh genomes. The frequency distribution is indicated by the curve width.

**Supplemental Figure 3: gp60 grouping in subgroups of genes.** Principal component analysis of genetic variation is shown. Points represent genomes and are color coded by gp60 grouping. Shapes represent patient location. The smallest dot represents the mixed infection from Bangladesh. Grouping is not statistically significant in these analyses. **A:** Only missense variants are shown. Missense variants are nonsynoymous and result in a change in amino acid sequence. **B:** Only missense variants in secreted proteins are shown and gp60 is excluded. Secreted proteins were identified by searching CryptoDB for ‘secreted’; these 95 genes have signal peptides but not all have known function. **C**: Principal component analysis of genetic variation using all SNPs and excluding the outlier sample from Bangladesh, identifier icddr,b 47.

**Supplemental Figure 4: Calculated recombination rate is influenced by the number of genomic sequences analyzed.** More *C. hominis* genome sequences were available in the previously published study, therefore we assessed the influence of study size on putative linkage disequilibrium [27]. Comparison between all *C. hominis* genomes (blue) and random subsets of 16-18 genomes (black) linkage disequilibrium decay, calculated with Plink 2.0.
