# Supplementary figures and images for: Genomic Heterogeneity of Cryptosporidium parvum Isolates From Children in Bangladesh: Implications for Parasite Biology and Human Infection
Source: J Infect Dis. 2023 Oct 13;228(9):1292–8. doi: 10.1093/infdis/jiad257 (PMC10629705; doi:10.1093/infdis/jiad257)

**Supplemental Table 1:** Summary of genomes used in this study.


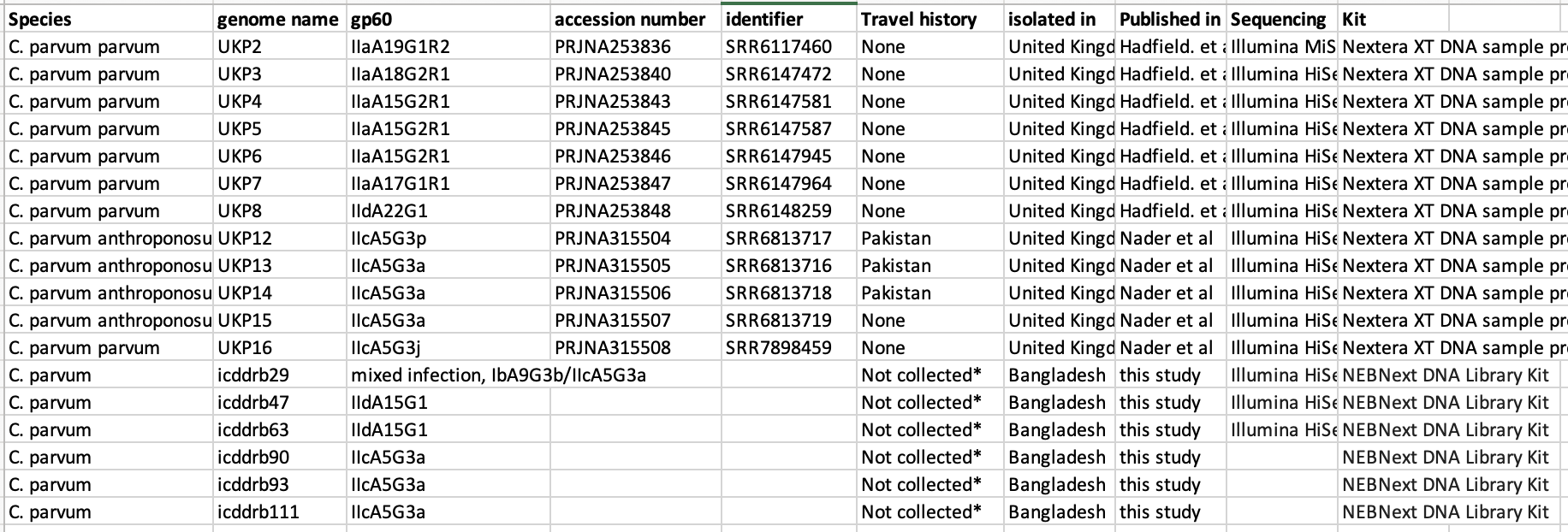

Supplement: jiad257_Supplementary_Data [file jiad257_supplementary_data.zip › Carey_Supplemental_Table_1.docx]

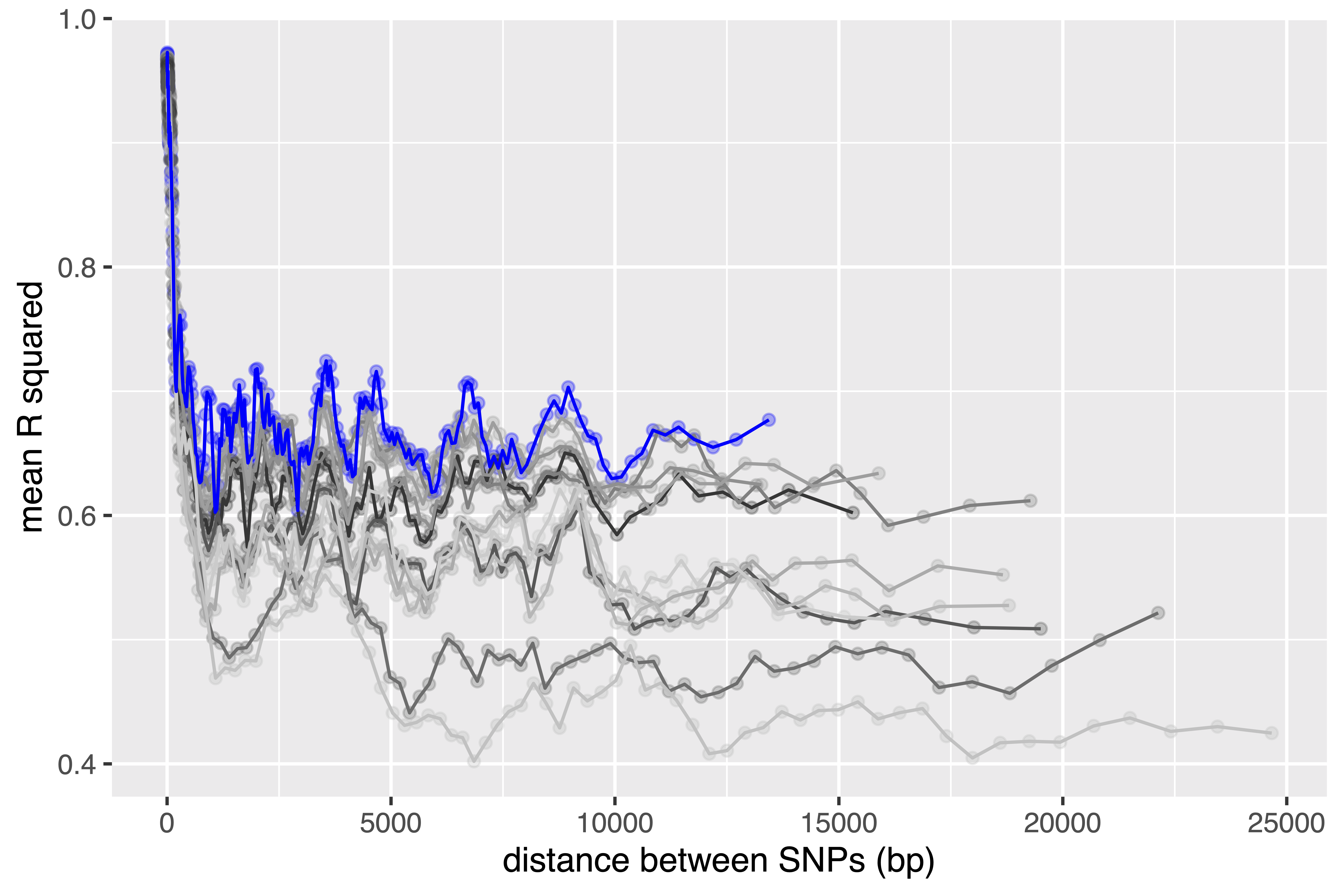

Supplement: jiad257_Supplementary_Data [file jiad257_supplementary_data.zip › SF4.tif]
